# Research and Remedies

**Published:** 1913-05-31

**Authors:** 


### RESEARCH AND REMEDIES.

### The Re-formation of Bone.

Two or three years ago Sir William Macewen startled the surgical world by some quite revolutionary ideas concerning the formation of new bone. His conclusions, based upon clinical observation checked and tested by animal experimentation, have been accepted far and wide as accurate; and evidence is constantly accumulating which tends to confirm his theories. In the *Edinburgh Medical Journal*, for instance, Dr. G. A. Pirie publishes some excellent *x*-ray photographs which show how completely the shaft of a long bone may be regenerated after excision for tuberculous osteomyelitis. In one of the cases he quotes, a perfect restoration of the limb was attained only after a lapse of two years; in another, the same result was obtained in a much shorter time. It was observed in one case that a very slow formation of new bone was changed into a rapid one after an accidental fall which fractured the new bone. Hence, Dr. Pirie suggests that whenever the process of repair is unduly slow, the bone should be purposely fractured, with the idea that this stimulus will at once hasten the manufacture of new bone and produce a useful limb.

### Wertheim's Results.

One of the most difficult tasks which surgeons and practitioners are called upon to face is the answering of the inquiries of the friends and relatives of patients in whom malignant disease has been diagnosed and operation for its cure advised. Surgical statistics have improved very greatly with respect to the real cure of most forms of malignant disease, and are now in fact far more favourable than most of the laity have any conception of. In the *American Journal of Obstetrics and Gynæcology* Professor Wertheim gives a full statistical account of the results of 500 operations for cancer of the uterus performed by him since he evolved the operative technique now known by his name. These statistics, as far as one can judge, have been honestly compiled, with no desire to obscure the whole truth or any part of it; and they are certainly of great interest not only because they deal with so large a number of cases, but also because they extend far enough back in point of time for an estimate to be possible of the final results of the operation. Before Wertheim introduced his operation, only about 15 per cent. of patients with cancer of the uterus could be operated on at all for cure; the other 85 were left to die, or at most a palliative operation was possible for them. Since 1898, however, only 519 out of 1,096 cases seen by Wertheim have been pronounced inoperable; and during the last few years the percentage of operable cases has risen to just over 60. Part of this remarkable improvement is no doubt due to earlier diagnosis; but the greater part of it must be set to the credit of Wertheim's operation. Out of the 15 per cent. operable in pre-Wertheim days, it is doubtful whether one-third (5 per cent. of the whole) were cured by the operations then in vogue; indeed probably 3 per cent. would be nearer the mark. What are the results of Wertheim's own operations? 607 cases were treated more than five years ago. Twenty-eight of these patients refused operation; three died of other diseases; thus 576 are left. Of these, 250 had Wertheim's operation performed; 63 died of the operation, directly or indirectly, 106 *have remained free from recurrence for more than five years*, and the remainder have relapsed. In other words, 18.4 per cent. of all cases seen have been apparently cured; and of those operated on and surviving the operation 57.6 per cent. are cured. There is reason to believe that the figures for the last five years when analysed will show further improvement.

